# Design and Testing of a Tool for Evaluating the Quality of Diabetes Consumer-Information Web Sites

**DOI:** 10.2196/jmir.5.4.e30

**Published:** 2003-11-27

**Authors:** Joshua J Seidman, Donald Steinwachs, Haya R Rubin

**Affiliations:** ^1^Center for Information TherapyHealthwise, IncWashington DCUSA; ^2^Department of Health Policy and ManagementJohns Hopkins UniversityBloomberg School of Public HealthBaltimore MDUSA; ^3^Departments of Medicine, Epidemiology, and Healthy Policy and ManagementJohns Hopkins School of Medicine and Bloomberg School of Public HealthBaltimore MDUSA

**Keywords:** Internet/standards, information management/standards, medical informatics/standards, guidelines, quality of health care, diabetes

## Abstract

**Background:**

Most existing tools for measuring the quality of Internet health information focus almost exclusively on structural criteria or other proxies for quality information rather than evaluating actual accuracy and comprehensiveness.

**Objective:**

This research sought to develop a new performance-measurement tool for evaluating the quality of Internet health information, test the validity and reliability of the tool, and assess the variability in diabetes Web site quality.

**Methods:**

An objective, systematic tool was developed to evaluate Internet diabetes information based on a quality-of-care measurement framework. The principal investigator developed an abstraction tool and trained an external reviewer on its use. The tool included 7 structural measures and 34 performance measures created by using evidence-based practice guidelines and experts' judgments of accuracy and comprehensiveness.

**Results:**

Substantial variation existed in all categories, with overall scores following a normal distribution and ranging from 15% to 95% (mean was 50% and median was 51%). Lin's concordance correlation coefficient to assess agreement between raters produced a rho of 0.761 (Pearson's r of 0.769), suggesting moderate to high agreement. The average agreement between raters for the performance measures was 0.80.

**Conclusions:**

Diabetes Web site quality varies widely. Alpha testing of this new tool suggests that it could become a reliable and valid method for evaluating the quality of Internet health sites. Such an instrument could help lay people distinguish between beneficial and misleading information.

## Introduction

Millions of people around the world are using the Internet each day to find health information, but they do so with little guidance regarding the actual accuracy and comprehensiveness of the information presented on the Web. The development and implementation of a valid method for evaluating the quality of Internet health sites could provide lay people with a tool to identify useful content more easily and to distinguish between beneficial and misleading information. Access to accurate and digestible information has the potential both to empower lay people and to raise the level of dialogue between them and their clinicians, thus enriching the patient-clinician relationship and ultimately improving the quality and efficiency of health care delivery.

This research sought to develop and test a health Web site evaluation model based upon a quality-of-care conceptual framework that evaluates information quality through performance measures, as well as structural measures that are proxies for information quality. The development of the conceptual framework is described in a previous paper [1]. In addition, this research provides a snapshot of the variability in the quality of diabetes consumer information on the Internet. The greater the variability that exists, the greater the need is for such evaluative tools. Previous research has demonstrated Web site variability in other areas [2] and the issues involved are discussed extensively in this issue of the Journal of Medical Internet Research [1].

## Methods

The methods involved in this research involve several levels. First, we explain the development of the model itself and the criteria used in evaluating health Web sites. Second, we discuss the sampling strategy options that could be used to select the subjects (Web sites) to be evaluated. Finally, we outline how the evaluation of individual Web sites was conducted.

### Proposed Model for Evaluating the Quality of Internet Health Information

For a tool to be systematic and objective, it needs to rely on elements that are valid and measurable. We have arrived at a set of criteria (Table 1) to include in a health-information Web site quality-evaluation tool through the lens of a quality-of-care conceptual framework and principles of qualitative meta-analysis. We examined both existing research available and tools that have been developed by health services researchers, physicians, Web experts, and medical librarians.

Although the set of criteria proposed above does not represent the entire universe of important aspects of health information, it does provide a reasonably-good cross-section of structural criteria and performance measures that can be assessed objectively. As described extensively elsewhere, structural measures address the underlying systems and infrastructure, whereas process measures assess the extent to which health care providers have done the right things. Structural characteristics include those in sections I, II, and III of Table 1: explanation of methods, validity of methods, and currency of information. Comprehensiveness (IV) and accuracy (V) serve as both performance and process measures of information quality in that they address how well the Web site performed in creating accurate and comprehensive (or high-quality) information against a set of criteria that were created based upon review of evidence-based practice guidelines and expert opinion.

There are undoubtedly other aspects of health-information quality and communication that affect quality of care. Certainly, user needs and expectations should be considered when evaluating information quality. Moreover, high-quality information by itself will not produce high-quality care, but it generally is a prerequisite for it.

To create valid measures of comprehensiveness and accuracy, we ideally would have compared the information available on Web sites to some gold standard, but no generic gold standard exists for overall health information. Therefore, the model focuses on one specific disease—diabetes—for which a reasonable gold standard exists, the American Diabetes Association's (ADA's) Clinical Practice Recommendations [3].

The Diabetes Quality Improvement Project (DQIP) [4] performance-measurement experience provides a useful model for developing and applying diabetes Web site information-quality performance measures, particularly with respect to content validity, a combination of face validity (or expert validity) and sampling validity. We initially extracted 20 elements to evaluate comprehensiveness and 10 specific criteria that relate to accuracy from the ADA's largely evidence-based practice guidelines. The comprehensiveness criteria reflected the breadth of content covered in the ADA guidelines, an important aspect of sampling validity. The ADA determined the coverage of topics based upon their expert panels' assessment of the clinical evidence. We added these 30 measures to a set of structural characteristics that were extracted from the existing tools and from suggested evaluation criteria in the literature. We wrote a definition for each item in the tool in order to precisely specify what would constitute a positive score on each criterion.

The next stage of measure development involved a review of measures by relevant experts for the purpose of strengthening the instrument's face validity. We sought feedback from 3 experts in diabetes performance measurement, all of whom served on the Diabetes Quality Improvement Project technical-expert panel (Barbara Fleming, MD, PhD; Sheldon Greenfield, MD; and Richard Kahn, PhD). Comments focused primarily on the comprehensiveness and accuracy sections, and can be grouped into 2 categories.

First, the experts believed that the set of comprehensiveness criteria was inadequate if it was to ensure that all major areas of diabetes care were addressed. Specifically, they suggested inclusion of 4 additional criteria in the comprehensiveness set (prevention, psychological aspects, neuropathy, and obesity), all of which were added to the tool, further strengthening the content validity of the tool. The experts were satisfied with the accuracy's section representative selection of items from the broader comprehensiveness set.

Second, one of the experts raised concerns about the feasibility of measuring accuracy based upon the measures' proposed definitions. That concern was addressed in 3 ways. First, the technical definitions for the accuracy measures received further refinement. Second, a reviewer-training session was added to improve the likelihood that the tool would be used according to objective criteria. Finally, actual testing of the proposed measures was conducted, just as it had been done prior to the approval of Diabetes Quality Improvement Project's performance measures.

In the reviewer-training session, we described each measure and technical definition to the 2 other reviewers (there were originally 2 external reviewers, but one dropped out later in the evaluation process, prior to reviewing any sites) and then went through a small sample of diabetes sites with the abstraction tool to demonstrate its application. Those initial reviews raised 7 specific questions, 4 of which related to the measure specifications in the accuracy section. These items were clarified and the guidance in Table 2 was provided to clarify the issues for each reviewer during their respective independent reviews. The external reviewer was a physician and a master's-degree candidate at the Johns Hopkins School of Public Health. This tool is designed to be applied by those with some public health background, but not necessarily with clinical experience; future assessment of the tool should examine the minimum skills required for reviewers.

**Table 1:** Proposed measurable criteria for credibility score for diabetes sites

**Category**	**Measurement**	**How Measured**
I. Explanation of methods	Content generation explanation Identification & disclosure	Site has explanation of process for generating its health content Author(s) listed and affiliations, credentials, and contact information provided
II. Validity of methods	Referenced material Peer review	Assertions supported by referenced material Material on site has gone through peer review
III. Currency of information	Updating process Content dating Timely update	Site has explanation of process for updating its health content Each Web page indicates date of last update Page updated within last 6 months
IV. Comprehensiveness of information	Screening Glycemia tests Nutrition Exercise Acute episodes Secondary diabetes Foot care Dyslipidemia Smoking cessation Nephropathy Retinopathy Immunization Insulin administration Oral medications Glucose monitoring Care of children Gestational diabetes DCCT (Diabetes Control & Complications Trial) implications UKPDS (United Kingdom Prevention of Diabetes Study) implications Insulin/glucose explanation Obesity Prevention Psychological aspects Neuropathy	Each of these aspects (primarily drawn from the clinical practice recommendations of the American Diabetes Association [3]) addressed and discussed on the Web site
V. Accuracy of information	Type 1 vs Type 2 Secondary causes Diagnostic tests HbA1c test Albumin tests Cholesterol tests Warning signs Hypoglycemia prevention Oral medications Rezulin	Explain Type 1 (lack of insulin) and Type 2 (insulin doesn't work effectively) Explain main secondary causes: drugs (pentamidine, corticosteroids, thiazides, niacin), pancreatic disease (chronic pancreatitis, hemochromatosis, cystic fibrosis, pancreatic surgery), endocrine disorders (Cushing's disease, acromegaly, pheochromocytoma, thyrotoxicosis), genetic syndromes (lipodystrophies, myotonic dystrophy, ataxia telangiectasia), insulin-receptor syndromes Explain diabetic threshold for fasting blood glucose test (> 125 mg/dL) and oral glucose tolerance test (> 199 mg/dL) Explain risk associated with HbA1c levels > 8%: impact on risk of coronary artery disease, kidney disease, and retinopathy Explain macroalbuminuria test (goal: negative) and microalbuminuria test (goal: < 30 mg/g creatinine) Explain HDL/LDL difference and LDL target level (< 100 mg/dL) Explain warning signs of acute diabetic episodes (fainting, seizures, state of serious confusion) Explain what brings on hypoglycemia (not eating enough/on time, exercise without food/insulin adjustment, weight loss, too much insulin/oral medications) Explanation of all 5 classes of oral medications (sulfonylureas, meglitinides, biguanides, glitazones, alpha glucosidase inhibitors) Explain liver problems associated with the glitazone Rezulin and why pulled back from market

**Table 2:** Issues identified in initial sample of diabetes sites during reviewer training*

**Issue**	**What to Do About It**
Many sites merely aggregate of miscellaneous information	Can still judge site by overall performance
Extent to which sites cover both childhood and adult diabetes	Sites specifically stating their focus on Type 1 diabetes are excluded; all others are included
Some structural criteria may be hard to assess, partially because some pages document structural issues well and other pages within the site may not	Judge based on whether the anchor site (main home page) documents structural characteristics, etc
Accuracy/Secondary Causes (V.b.) measure: Some sites may address some, but not all, of the causes	Score positive if they include at least 4 of the 5 causes
Accuracy/Albumin Tests (V.e.) measure: Some sites may use "proteinuria" instead of "macroalbuminuria"	Either "proteinuria" or "macroalbuminuria" is fine
Accuracy/Hypoglycemia (V.h.) measure: Some sites may address some, but not all, of the prevention methods	Score positive if they include at least 3 of the 4 prevention methods
Accuracy/Oral Medications (V.i.) measure: Some sites may refer to acarbose rather than the broader drug class name of alpha glucosidase inhibitors	Score positive if either term is used

^*^ Roman numerals plus letters (V.b., V.e., V.h., and V.i.) refer to Table 1.

### Sampling Strategy

We selected a specific search term (ie, "diabetes") and used the Direct Hit search engine (now subsumed by the Teoma search engine) [5], which claims that it tracks the most "popular" sites by search term. Any sites coming from a duplicate parent were eliminated, as they were covered in the review of the parent site (eg, *www.diabetes.com* would include any pages that include *www.diabetes.com/xxx*). We also developed a standardized set of eligibility criteria. Sites were excluded for 4 reasons. First, sites addressing only Type 1 diabetes or "juvenile diabetes" were excluded because some of the comprehensiveness criteria would not apply to Type 1. Second, a site in which there was a clear explanation that it was not designed for consumers would not be appropriate for an evaluation of consumer health Web sites. Third, sites that only included "news" and were not designed to offer general diabetes content were not evaluated. Finally, sites were excluded if the Web site address led to a dead link.

### Evaluation Process

With the final tool for evaluation of Web site credibility, we began the process of evaluating the sites that met the eligibility criteria through an objective and systematic process.

First, we created a data-abstraction tool, which includes all of the proposed evaluation criteria (listed in Table 1) as well as additional background or "demographic" data on the individual Web sites. This demographic data was used to characterize Web sites, primarily with respect to sponsorship characteristics (advertising vs no advertising, profit vs not-for-profit, academic vs nonacademic, and governmental vs private). The abstraction tool and evaluation-definitions table were accompanied by instructions (originally clarified in a table sent via e-mail to the reviewers) on specific items that arose during the reviewer-training session (which are summarized Table 2).

Second, we created a set of composite scores by section and overall score based upon the evaluation instrument and the data-abstraction tool.

Third, we used the software application "Catch the Web" [6] to "freeze" (download a copy of) Web sites.

Finally, the external reviewer and the principal investigator (JS) scored each site with respect to the attributes in the evaluation model. The Web site received 1 point for each criterion that it met (eg, 1 point if it explains its process for generating health content [I.a., in Table 1], 1 point for conducting a peer-review process [II.b., in Table 1], and so forth). The same held true for the comprehensiveness criteria. For the accuracy criteria, however, the site only was evaluated (and therefore only counted in the denominator) on those aspects that it did address, thus maintaining a distinction between accuracy and comprehensiveness. Otherwise, a site would get penalized twice for not providing information on kidney disease testing, when it really only represents a failure of comprehensiveness or breadth, rather than the provision of inaccurate health information.

### Analysis of the Evaluation Tool

Assessment of the tool involved an evaluation of the tool's feasibility, performance on individual criteria, distribution of scores, and reliability. Feasibility depends on how long it takes to review sites (quantitative) and whether reviewers had trouble applying the instrument (qualitative).

Considerable controversy exists in the literature regarding selection of statistical methods for assessing reliability in the development of new tests, tools, and indexes. Most of this debate relates to measures of clinical evaluation, and no research has addressed this issue for the tool being tested here.

We employed 3 methods to test inter-rater reliability. First, we used the kappa statistic to assess how much agreement existed between reviewers relative to expected agreement by chance on each criterion. The kappa value is influenced substantially by "prevalence" so that rare events are likely to have low kappas even when agreement is high [7]. To address this limitation, a second measure of reliability, Lin's concordance correlation coefficient, was used to measure how close the 2 raters' judgments fall along a 45-degree line from the origin (or a slope of exactly 1.00) [8]. Additional data are presented for Pearson's r, a direct test of correlation. For the reasons described above, Lin's concordance correlation coefficient appears to be the most-appropriate method for evaluating the overall reliability of our index, but it is worthwhile to examine the kappa values of each item in the index—particularly in this alpha-testing phase—to provide future researchers clear targets for index refinement.

## Results

Of the 90 sites selected from November 2001 through January 2002, the external reviewer examined 69 and the principal investigator (JS) reviewed 21, plus both reviewed 30 sites for reliability testing.

### Assessment of the Evaluation Tool

Assessment of the Diabetes Quality of Internet Information (Diabetes QII) tool involves several components: feasibility, score means, distributional properties, reliability, and individual criterion performance.

#### Feasibility

The mean time required to review each site was 30.26 minutes, including identifying sponsorship characteristics, process measures, and outcome measures. Time to review ranged from 3 to 75 minutes, with a standard deviation of 16.26 minutes. The level of variation reflects the diversity in the quantity of information that needed to be reviewed on each Web site.

Qualitatively, some of the information was difficult to locate, although this was much more problematic for the process measures than the outcome measures. In addition, in some cases, trying to discern sponsorship characteristics was difficult and time-consuming. Since sponsorship is not integral to quality measurement, some time could be saved by dropping this item.

#### Distribution of Scores and Performance Summary

There was considerable variation in the different scoring sections and wide variation in performance overall, with a mean of 50% and a median of 51%. Appendix 1 presents the 90 sites in order of overall score (and secondarily by outcome score) with scoring section breakdowns. There was also great variability among sites in all categories of scores (see Table 3). Overall scores ranged from 15% to 95%, comprehensiveness scores from 13% to 96%, and performance composite scores (combining accuracy and comprehensiveness) from 14% to 97%. The accuracy composite score and the process measure composite score (the latter being a combination of explanation of methods, validity of methods, and currency of information) each ranged from zero to perfect (0% to 100%).

**Table 3:** Distributions of scores for 5 categories

	**Structure Composite**	**Overall Score**	**Performance Composite**	**Comprehensiveness**	**Accuracy**
Smallest	0%	15%	14%	13%	0%
5th percentile	0%	23%	21%	21%	0%
10th percentile	0%	28%	31%	31%	15%
25th percentile	0%	38%	41%	46%	30%
Median	29%	50%	55%	58%	43%
75th percentile	57%	65%	70%	75%	63%
90th percentile	71%	75%	79%	83%	78%
95th percentile	86%	80%	82%	88%	78%
Largest	100%	95%	97%	96%	100%
Mean	31%	51%	56%	59%	44%
Interquartile range	57%	27%	29%	29%	33%
Standard deviation	28%	18%	19%	20%	23%
Skewness	0.597	0.154	-0.127	0.328	0.039
Kurtosis	2.295	2.352	2.441	2.321	2.640
Shapiro-Wilk w test *P*	< .001	.44	.65	.17	.56
Assessment of normality	Not normal	Normal	Normal	Normal	Normal

Mean scores were each within 10 percentage points of 50%, except for the structure measure composite score (mean 31%). The mean of the overall score was 51%; of the performance composite, 56%; comprehensiveness, 59%; and accuracy, 44%. The medians were similar to the means: 29%, 50%, 55%, 58%, and 43%, respectively. The interquartile ranges were 57%, 27%, 29%, 29%, and 33%, respectively.

#### Instrument Reliability

**Figure 1:**
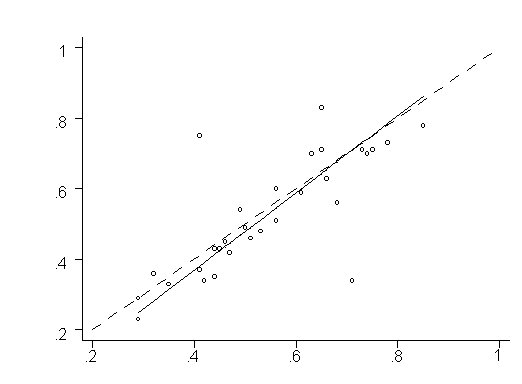
Graphical presentation of Lin's concordance correlation coefficient*

Lin's concordance correlation coefficient produced a rho of 0.761 with a standard error of 0.079. See Figure 1 for a graphical presentation of the data (in which the goal is to have a slope of 1.0 from intercept at 0). The Pearson's r was similar, at 0.769. This set of values suggests moderate to high agreement between raters.

* Lin's concordance correlation coefficient:

Data must overlay dashed line for perfect concordance.

Observations = 30, rho = 0.761 (95% CI, 0.607-0.916), standard error (rho) = 0.079, *P*< .001

Pearson's r = 0.769

Slope = 1.094, intercept = -0.069

The kappa statistics for the individual criteria varied substantially, from a low of -0.0465 to a high of 0.7826, with an overall average just under 0.40 (see Appendix 2). Forty-four percent (15 of 34) of the performance composite criteria had kappa values over 0.50 and 68% of them (23 of 34) had values that were statistically significantly different from the expected level of agreement.

A number of the low kappa values occurred in spite of high levels of agreement on those particular items (see "Methods" section for an explanation by Feinstein and Cicchetti [7] regarding why this paradox occurs). For example, the 2 worst kappa values—nutrition/comprehensiveness (-0.0465) and secondary causes/accuracy (0.0000)—had high levels of agreement (90.00% and 96.67%, respectively) but also had exceptionally-high levels of expected agreement because the criterion did not prove to differentiate among sites well.

#### Individual Item Performance

There was great variation in the scores of individual items, as presented in Table 4, suggesting that different criteria measure different aspects of Web site quality. The median and mean are 51.11% and 51.66%, respectively, and the standard deviation is 25.73%. No items have averages below 5% or above 95%. Although they range from 7.78% to 91.11%, more than 80% of the items average between 15% and 85% (the 10th percentile is 15.56% and the 90th percentile is 85.56%).

**Table 4:** Individual item performance

**Criterion**		**Percentage of Web Sites Scoring Positively**
**Process measures average**		30.63
	Content generation explanation	30.00
	Identification and disclosure	46.67
	Referenced material	34.44
	Peer review	26.67
	Updating process	10.00
	Content dating	51.11
	Timely update	15.56
**Comprehensiveness average**		**59.21**
	Screening	38.89
	Glycemia tests	74.44
	Exercise	85.56
	Acute episodes	63.33
	Secondary diabetes	30.00
	Foot care	71.11
	Dyslipidemia	64.44
	Smoking cessation	42.22
	Nephropathy	91.11
	Retinopathy	88.89
	Immunization	7.78
	Insulin administration	64.44
	Oral medications	74.44
	Glucose monitoring	75.56
	Care of children	26.67
	Gestational diabetes	70.00
	DCCT (Diabetes Control & Complications Trial) implications	41.11
	UKPDS (United Kingdom Prevention of Diabetes Study) implications	21.11
	Nutrition	90.00
	Insulin/glucose explanation	71.11
	Prevention	41.11
	Psychological aspects	27.78
	Neuropathy	82.22
	Obesity	77.78
**Accuracy average (of those sites addressing item)**		**48.24**
	Type 1 vs Type 2	78.65
	Secondary causes	22.22
	Diagnostic tests	66.67
	HbA1c test	55.88
	Albumin tests	15.19
	Cholesterol tests	35.59
	Warning signs	87.27
	Hypoglycemia prevention	68.97
	Oral medications	39.71
	Rezulin	12.22
**Outcome composite average**		**55.98**
**Overall average**		**51.66**

## Discussion

### Great Variability in Quality of Internet Diabetes Information

The wide variation in scores demonstrates that considerable variation exists in the quality of consumer diabetes information on the Internet. In addition, the overall mediocre Web site performance (average score of 50%) suggests that the level of inaccuracy and missing information is substantial. This relatively-low Web site quality suggests that consumers need a way to discern which sites offer high-quality information.

The tool also appears not to suffer from floor or ceiling effects in that there is variation even among "poor" performers as well as room for improvement. There were no overall scores of either 0% or 100% and few that were that close to either end of the spectrum. The fifth percentile was 23% for overall scores and 21% each for the comprehensiveness score and the performance composite. Only 5% of sites received a score of 80% or better on either the overall or outcome composite scores, suggesting room for improvement. One might expect that institutionalization of a Web site information-quality measurement system might lead to longitudinal improvement on scores and reduction in variation, as has been the case with HEDIS (Health Plan Employer Data and Information Set) measurement and health plan performance [9]. For example, the percentage of members in reporting health plans receiving a prescription for beta blockers after a heart attack has steadily increased since the measure was introduced, from a median of 64% in 1996 to 92% in 2000 [10]. If the measures are a valid representation of quality, then one can make the argument that the competitive performance measurement approach has driven system-wide quality improvement.

What is the impact of poor performance? For failed prescription of beta blockers, the evidence suggests that there is no doubt that some people will die due to poor adherence. One could argue that similar risks are involved in the case of inaccurate or misleading Internet health information. According to a January 2002 Pew Internet & American Life Project survey [11], 15 million Americans used the Internet to make a health care decision in the years 2000-2001. As more consumers determine treatment choices based on what they (or their families) read on the Web, the impact of bad information will grow. In the case of diabetes, inaccurate information could mislead a consumer into failing to be aware of all of the signs that an acute diabetic event is beginning. Incomplete information could suggest to the lay person with Type 2 diabetes that limiting carbohydrate intake (to moderate blood sugar levels) is sufficient dietary guidance, when he or she is actually most likely to die from a cardiovascular event, for which fat intake may be equally (or more) important.

The major practical implementation challenge relates to making sure that the tool is generalizable from one condition, diabetes mellitus, to the vast array of medical and health care topics. Nothing from this research demonstrates the quality of Web sites for any condition other than diabetes. In fact, many of the sites—including 3 of the top 5—are diabetes-specific sites, so one would not expect to seek information from them about other diseases. The sample from which to choose for breast cancer, liver disease, or schizophrenia undoubtedly would be much different. However, the intrinsic nature of a tool that addresses performance measures of information quality is that it focuses on a particular condition, especially in the domain of comprehensiveness.

### Validity of the Tool

As discussed earlier, testing the validity of a tool in an area where no other research exists is a considerable challenge. Nevertheless, some aspects of validity have been addressed. Deriving the original measures from the wide range of ADA evidence-based practice guidelines provided some degree of sampling validity. The face validity of the tool was addressed by having the tool reviewed by 3 diabetes performance-measurement experts and then making adjustments to the tool based upon their suggestions. Further refinements of the tool should involve an iterative process with these experts (and additional experts who bring other perspectives, such as diabetes nurse educators and consumers) for 2 reasons. First, the experience of implementation might inform experts' opinions about the value of individual criteria, thus creating an opportunity to combine the quantitative findings with a consensus process to make the tool more efficient and precise. Second, expert input is important to ensure that alterations to the tool based upon quantitative findings do not undermine its face validity. For example, item reduction based upon quantitative aspects of validity could eliminate items so central to the understanding of diabetes information quality that the tool could become less valid.

The tool's ability to differentiate among sites and its lack of floor and ceiling effects offers other suggestions of validity. Further exploration with diabetes measurement experts can be used to ensure that those differences reflect actual distinctions in information quality.

Given that each site evaluation took just over a half hour, the tool does not appear to be particularly burdensome to implement for a single disease. Furthermore, some of that time included the effort to identify each Web site's sponsorship characteristics for the purposes of this research, which would not be part of the evaluation tool itself. In addition, one might anticipate that greater experience with the tool might improve efficiency in the evaluation process. If someone is trying to find an objective, systematic approach to evaluating the quality of diabetes information on the Internet, this is a reasonably efficient and practical solution.

### Tool Reliability and Opportunities for Improvement

The major test of reproducibility, inter-rater reliability, produced good results but also suggested specific opportunities for improvement. The test of concordance (Lin's correlation concordance coefficient) and Pearson's r produced almost identical results: 0.761 and 0.769, respectively. Depending upon which statistician's guidance one chooses to use, this level of agreement could be characterized as "excellent" [12], "good" [13], "substantial" [14- 15], or "moderate" [16].

Setting aside the argument of whether the reliability of the tool tested was moderate, excellent, or somewhere in between, the more-important finding is that the experience of alpha testing this tool has suggested several ways in which reliability could be improved.

First, as the graphical plotting of Lin's concordance correlation coefficient shows in Figure 1, there are 2 clear outliers, which turn out to be Diabetes Education Network (principal investigator, JS, at 75% and external reviewer at 41%) and Diabetes Australia (principal investigator, JS, at 34% and external reviewer at 71%). When the 2 outliers are excluded from the data set, an analysis of the 28 remaining pairs shows a rho of 0.924 and a Pearson's r of 0.932 (a level that suggests excellent rater agreement), a difference of 0.163 on both reliability measurements. A postanalysis discussion between the 2 raters revealed some issues with these 2 sites that could be addressed by refinement of the tool and reviewer training criteria.

In both cases, these sites produce little to no consumer content of their own. They each include many links to other sources—either non-consumer-oriented (eg, Australian diabetes practice guidelines for professionals) or external—some of which were erroneously not captured during the original Web site freezing process. According to the reviewer instructions, Web pages not frozen at the time of abstraction should not be included in that site's evaluation because they may not have been there with precisely the same content at that time. However, it appears that this may not have been adhered to for these 2 sites.

In a dynamic Web site reviewing atmosphere, in which Web site review did not need to be based on the content posted on a site at a specific moment in time, this situation may not have occurred because no freezing software would need to be used. The reviewer instructions—both written and during training—could be made clearer regarding guidance for linked sites. In particular, further clarification could be made regarding the inclusion of links to nonconsumer (professional) content, such as provider practice guidelines.

The second way to improve inter-rater reliability for future versions of the tool relates to the specifics of the reviewer training sessions. Although we conducted a reviewer training session, there is no way to assess if it was thorough enough. Now, with the experience of having done this once, we would add and modify elements of that training. Such training enhancement likely would improve inter-rater reliability, and thus ensure that the tool could be applied more reliably in an accreditation or evaluation system in the real world.

Third, experience with the tool has also suggested elements of it that could benefit from clearer definitions. Precise, technical specifications are a critical element of any quality measurement system, but such definitions typically are finalized following field testing of an instrument. Better specifications could improve the reliability and validity of the tool in the future.

Fourth, experience with the tool has also demonstrated that "accuracy" and "comprehensiveness" may not be entirely distinct. In some cases, the inaccuracies were not entirely "wrong." For example, a site that discussed hypercholesterolemia as a complication of diabetes received a positive score on that criterion in comprehensiveness and therefore was scored (in the denominator) on that item in the accuracy section. If that site then failed to explain the different types of cholesterol and the appropriate low-density lipoprotein (LDL) target levels, it did not receive a positive score in accuracy, despite the fact that no "erroneous" information was presented. Some might argue that this is more a failure of comprehensiveness than accuracy, whereas the site that explains LDL but suggests the wrong target level is scored in the same way under this tool. Further research to refine the scoring system of this tool would be useful (see "Future Research Directions" section below).

Finally, evaluating international sites was a challenge because some of the recommendations may be different in other countries due to different standards of practice. For example, one of the most-basic issues in diabetes is defining what constitutes a diagnosis of the condition. The World Health Organization definition relies on a fasting blood glucose threshold of 140 mg/dL, whereas ADA—the accepted standard in the United States—uses a more-aggressive target of 126 mg/dL. Ultimately, we decided to include foreign-sponsored sites in the analysis under a US-developed system because this is an evaluation primarily for use by people in the United States and global access to different sites means that it is just as easy for an American to look at the DiabetesAustralia.com Web site as the ADA's Web site. However, because our review was being conducted concurrently with that of the external reviewer, that judgment was applied inconsistently between the 2 reviewers.

With alpha testing concluded, a beta test that addressed the issues above could vastly improve the inter-rater reliability, a key attribute of future successful implementation of any tool designed to offer an objective, systematic method. In addition, it may be worthwhile to consider eliminating, amending, or replacing items for which the kappa statistic was not statistically significant, which included 2 process criteria (updating process and timely update), 7 comprehensiveness criteria (exercise, acute episodes, foot care, dyslipidemia, care of children, prevention, and obesity) and 4 accuracy measures (Type 1 versus Type 2, secondary causes, cholesterol tests, and warning signs).

Examination of scores by evaluative section reveals some additional interesting findings. Comprehensiveness scores were substantially higher (58% and 59% median and mean, respectively) than accuracy scores (43% and 44%). This finding differs from the RAND/CHCF study [17] that evaluated "coverage" and "correctness," and found that Web sites were more likely to be accurate than to cover the clinical terrain comprehensively. As stated above, one of the areas for further clarification in this tool is the distinction between comprehensiveness and accuracy. The difference in terminology between the Diabetes Quality of Internet Information tool and the RAND tool also may be more than simply a semantic distinction. RAND's goal of assessing "correctness" perhaps speaks more directly to the distinction between erroneous and correct information. In contrast, "accuracy" is a broader goal that relates more to the degree of specificity of the information provided in helping consumers to understand a condition and change behaviors. Because our tools address different clinical conditions, a more thorough comparison of the individual criteria is difficult.

There was little correlation among the various criteria. One would anticipate that this type of index would have criteria independent of each other. Perhaps what was surprising was how few criteria had correlations of 0.50 or higher. Out of 820 possible correlations, only 12 had at least this modest correlation (contact author for a correlation matrix). None of the structural measures correlated at this level with any of the accuracy or comprehensiveness characteristics. Three of the structural measures were correlated with each other (content generation explanation, identification and disclosure, and peer review) in the 0.55 to 0.65 range. The only other correlations above 0.60 between any 2 of the comprehensiveness or accuracy criteria were the comprehensiveness criteria of retinopathy and nephropathy (at 0.76), and neuropathy and nephropathy (at 0.67); neuropathy and retinopathy were modest as well (0.58). Given these correlations, one might also expect that other complications in the comprehensiveness section would be somewhat high as well (eg, foot care and dyslipidemia), but none of the correlations among other criteria were higher than the 0.40 range.

### Limitations

Limitations that could have affected the results of this research fall primarily into 2 categories: sample and search strategy, and site review and evaluation.

#### Sample and Search Limitations

The sampling had 3 limitations.

First, as described in the methods, the goal of the search strategy was to identify the most-popular sites for diabetes information, the rationale behind the selection of the Direct Hit search engine. However, there is no guarantee of Web site popularity because Direct Hit considers its search algorithm proprietary and therefore does not make it available for public critique.

Second, the popularity of some developers of Web site content may not be accessible through standard search engines, particularly with respect to information products licensed by content companies to consumer portals.

Third, the goal to freeze sites at a single point in time was not successful. It was time-consuming to freeze each individual page, a factor unrelated to the feasibility of the tool because the freezing was for research purposes rather than an intrinsic part of the evaluation system. This freezing process took many weeks to complete, thus eliminating its potential benefit. In retrospect, it would have been more efficient to go immediately to the evaluation phase of the research. The fact that some sites were frozen in November 2001 and others in January 2002 could affect the situational reliability of the evaluation, as some of the data could have changed. However, it is unlikely that this would have substantially changed the results. In the future, it may be valuable to do the opposite; that is, given the dynamic nature of the Web, it would be worthwhile to know how well sites update themselves to reflect new scientific information.

#### Review and Evaluation Limitations

There were 6 limitations of the Web site review and evaluation.

First, because this research only addresses diabetes, one cannot generalize these findings to other aspects of health information.

Second, since no attempt was made to blind Web site names (it would have been too time-consuming for the purposes of this research), it is possible that reviewers' personal biases could have affected the evaluation scores.

Third, there was only one external reviewer. Therefore, the data included in this overall analysis also derive from the principal investigator's (JS's) evaluations. In order to minimize bias at the upper end of performance (since the top-scoring site—Healthwise—employs one of the authors), we only used the scores of the external reviewer for the top-performing sites.

Fourth, the study used the site's own description of its activities to determine the independent variables, which were not clear in all cases. One might think that the extensions of the Web sites (eg, .com, .org, .gov, and .edu) would provide much of that information, but there are many examples of instances where they are misleading. Many sites with .com extensions are not-for-profit. State and foreign government sites do not use .gov. Some state government-sponsored Web sites are "housed" in academic institutions that have .edu extensions. In addition, the myriad subsidiary arrangements sometimes make it difficult to discern for-profit and not-for-profit status, as some for-profit companies have nonprofit subsidiaries and vice versa.

Fifth, the criterion of "timely update" used an arbitrary time cut-off of 6 months. The rationale was to create some time cut-off to separate those sites that update their content regularly from those that do not. However, there is little reason to suspect that a site updated 26 weeks ago is better than a site updated 27 weeks ago.

Finally, the Internet is changing rapidly and is a moving target. Just as the state of the Internet has changed dramatically since this research began, many other changes can be expected in the near future that could change some of these findings.

### Future Research Directions

Future related research would be helpful in 2 areas: refinement of the existing diabetes tool and application of the diabetes tool to other conditions.

First, refinement of the existing tool primarily relates to addressing the issues raised in the reliability section above. More-precise technical specifications of the review criteria, more-thorough reviewer training, and a clearer distinction between accuracy and comprehensiveness would lead to an improved second version of the tool. A reexamination of the tool by the diabetes performance-measurement experts or an expert panel could allow the tool to provide even more differentiation among sites, particularly in the comprehensiveness section where there was less variation in scores. With that work completed, additional methodological research should be done on the index construction itself, as outlined above.

Second, with respect to the need for research on Web site evaluation tools for other conditions, one of the critical factors is dealing with varying degrees of an evidence base across diseases. Whereas treatment for diabetes has a relatively-strong evidence base—and some, like cardiovascular disease, probably are even stronger in that respect—other conditions have much more limited evidence (or it changes rapidly) on which a Web site can base its information. This has implications for criteria selection in terms of both what should be covered on a Web site (comprehensiveness) and precisely what the site should say (accuracy).

### Conclusions

There is wide variation in the accuracy and comprehensiveness of online diabetes information and no existing mechanism for consumers to get detailed, objective information about true Web site quality. Furthermore, this research also demonstrates the limited utility of using proxies such as sponsorship characteristics to help guide consumers in searching for health Internet information.

This research also highlights the alarming amount of inaccurate and incomplete Internet information on diabetes. Given the increase in consumer use of the Web to make health care decisions, the potential threats to patient care are substantial. If diabetes information is incomplete, a consumer may not be aware of all the various complications of diabetes and thus not know to get tested for certain conditions. If a consumer finds inaccurate information on the Web, he or she may not be aware, for example, of the symptoms that indicate the onset of an acute diabetic event.

Objective review of performance in producing health information quality, expressed in terms of accuracy and comprehensiveness of information, can offer consumers a tangible and useful tool in navigating the online health universe.

## Abbreviations

ADA: American Diabetes Association

## Appendix 1

### Scoring by section; ordered first by overall score and second by outcome total.

**Table A1:** Scoring by section; ordered first by overall score and second by outcome total

**Web Site**		**Scoring Section**				**Overall Score, %**
**Name**	**URL**	**Process Total, %**	**Outcome Total (Outcome Score), %**	**Comprehensiveness, %**	**Accuracy, %**	
Healthwise*	http://www.healthwise.org/p_demos	86	97	96	100	95
Diabetes Living	http://www.diabetesliving.org/	57	97	96	100	90
Canadian Diabetes Association	http://www.diabetes.ca/	86	88	92	80	88
American Diabetes Association	http://www.diabetes.org/	86	85	88	78	85
MayoClinic.com	http://www.mayoclinic.com/	71	82	92	60	80
Wisconsin Department of Health	http://www.dhfs.state.wi.us/health/diabetes	71	79	88	56	78
Helios Health	http://www.helioshealth.com/diabetes	86	76	79	67	78
DrKoop.com	http://www.drkoop.com/	100	70	79	44	75
MEDLINEplus	http://www.nlm.nih.gov/medlineplus	57	49	83	67	75
South Dakota DHHS	http://www.sddiabetes.net/	57	78	79	75	74
Diabetes Insight	http://www.diabetic.org.uk/	29	82	83	78	73
Joslin Diabetes Center	http://www.joslin.harvard.edu/	29	82	83	78	73
Health A to Z	http://www.healthatoz.com/atoz/Diabetes	57	76	75	78	73
Net Doctor	http://www.netdoctor.co.uk/	56	76	79	70	73
Canadian Medical Association Journal	http://www.cma.ca/cmaj	71	74	83	50	73
Diabetes Australia	http://www.diabetesaustralia.com.au/	71	71	83	29	71
University of Massachusetts Medical School Diabetes Handbook	http://www.umassmed.edu/diabeteshandbook	43	73	83	44	68
University of Pennsylvania Health System	http://www.uphs.upenn.edu/health	57	70	71	67	68
South Carolina Diabetes Association	http://www.musc.edu/diabetes	14	77	75	86	66
Diabetes News	http://www.diabetesnews.com/	0	79	83	67	65
Diabetes Mall	http://www.diabetesnet.com/	14	76	75	78	65
NIDDK	http://www.niddk.nih.gov/	57	67	71	56	65
Texas Department of Health	http://www.tdh.state.tx.us/diabetes	57	67	75	44	65
Focus on Diabetes	http://www.focusondiabetes.com/	71	64	63	67	65
WebMD	http://my.webmd.com/index	29	71	75	60	63
Colorado Health Net	http://www.coloradohealthnet.org/diabetes	29	70	75	56	63
Diabetes Guidelines Europe	http://www.staff.ncl.ac.uk/philip.home	43	67	67	67	63
National Service Framework for Diabetes	http://www.doh.gov.uk/nsf/diabetes	57	64	75	33	63
International Diabetes Institute	http://www.diabetes.com.au/	14	72	75	63	62
Diabetic Retinopathy	http://medweb.bham.ac.uk/easdec	43	66	71	50	62
Diabetes Program of Ohio	http://www.diabetesohio.org/	29	68	88	20	61
Online Med Info	http://www.onlinemedinfo.com/	14	70	67	78	60
Virginia Mason	http://www.virginiamason.org/	29	66	74	38	59
Diabetes Scene	http://www.banting.com/	43	63	63	63	59
Doc Guide.com	http://www.docguide.com/	0	68	79	40	56
Annenberg Center for Health Services	http://www.annenberg.org/achs	29	63	67	50	56
Diabetic Digest	http://www.thediabeticdigest.com/	29	62	75	30	56
Merck Manual	http://www.merck.com/pubs/mmanual_home	57	56	67	30	56
Diabetic.com	http://www.diabetic.com/	0	68	67	61	55
Healing Well	http://www.healingwell.com/	29	58	63	44	53
Pancreatic Diseases	http://66.70.75.130/bin/ctwe	86	45	46	43	53
Oregon Department of Health	http://www.ohd.hr.state.or.us/diabetes	71	47	42	67	51
Diabetes Mellitus	http://www.diabetes-mellitus.org/	0	61	67	64	50
BBC	http://www.bbc.co.uk/health/diabetes	14	58	63	43	50
Lilly Diabetes	http://www.lillydiabetes.com/	29	55	58	44	50
Medical College of Wisconsin	http://healthlink.mcw.edu/article	71	45	50	29	50
Diabetes Institutes Foundation	http://www.dif.org/	14	57	63	33	49
About.com	http://www.diabetes.about.com/	14	56	58	50	49
Novo Nordisk	http://www.diabetesdiary.com/	43	50	54	38	49
Yahoo	http://www.yahoo.com/health/diseases	14	55	58	44	48
International Diabetes Foundation	http://www.idf.org/	0	28	67	29	47
East Texas Medical Center	http://www.etmc.org/diabetes	14	53	58	38	46
Defeat Diabetes	http://www.defeatdiabetes.org/	29	48	58	22	45
Mamas Health	http://www.mamashealth.com/diabetes3	0	53	58	38	44
Black Women's Health	http://www.blackwomenshealth/diabetes	29	47	58	13	44
Reverse Diabetes	http://www.reversingdiabetes.org/	0	53	58	33	43
Medical Data	http://www.medicaldata.com/	14	50	54	33	43
Eastern Virginia Medical School	http://www.evms.edu/diabetes	29	46	50	25	43
Diabetes News on the Net	http://www.diabetesnewsonthenet.com/	0	52	54	43	42
University of Michigan	http://www.med.umich.edu/1libr/topics/diabetes	0	52	58	29	42
New Zealand Guidelines Group	http://www.nzgg.org.nz/library	43	41	46	20	42
Diabetes Education Network	http://www.healthtalk.com/den/index	0	50	46	67	41
Diabetes at Mercy Medical Center	http://www.diabetes.mdmercy.com/	0	50	54	33	41
International Diabetes Center	http://www.idcpublishing.com/	0	50	54	38	41
Dr. Mirkin	http://www.drmirkin.com/diabetes	57	38	46	13	41
4Women.gov	http://www.4woman.gov/faq/diabetes	14	45	50	33	40
Endocrinologist.com	http://www.endocrinologist.com/diabetes	0	48	54	29	39
Diab Care	http://www.diabcare.de/diabetes	0	47	54	25	38
Utah Diabetes Control Program	http://www.health.state.ut.us/cfhs	0	43	50	17	35
CDC	http://www.cdc.gov/diabetes/index	43	33	33	33	35
Diabetes Therapies.com	http://www.diabetes-therapies.com/	0	41	42	38	33
Solaris	http://www.solarishs.org/diabetes	0	41	42	38	33
Endocrine Web	http://www.endocrineweb.com/diabetes	14	38	33	60	33
IDD Trust International	http://www.iddtinternational.org/	14	38	33	60	33
Paralumun	http://www.paralumun.com/diabetes	0	39	38	43	32
University of Minnesota Diabetes Institute	http://www.diabetesinstitute.org/	0	39	42	25	31
BD Diabetes	http://www.bddiabetes.com/	14	36	42	0	31
American Podiatric Medical Association	http://www.apma.org/topics/Diabetes	0	37	38	33	29
Tin Man	http://www.tinman.com/diabetes	0	37	38	33	29
Diabetes and CAD	http://www.chebucto.ns.ca/Health/CPRC/diabetes	14	32	29	50	29
Your Health Your Hands	http://www.yourhealthyourhands.com/diabetes	29	30	33	0	29
Diabetes Control Center	http://www.dr-diabetes.com/	29	28	25	40	28
Diabetes Foundation of Mississippi	http://www.msdiabetes.org/	0	33	38	17	27
Healthy Wave-Diabetes	http://www.healthywave.com/healthbeat/diabetes	29	22	25	0	24
University of Manitoba	http://www.umanitoba.ca/outreach/drtc	43	19	21	0	24
FIT Foundation	http://www.diabetestrends.com/	29	21	25	0	23
Medical Library of Utah	http://www.medlib.med.utah.edu/	43	14	17	0	20
South Carolina Diabetes Prog	http://www.musc.edu/diabetes	14	19	13	67	18
Family's Guide to Diabetes	http://diabetes.cbyc.com/	0	21	17	50	17
Geocities.com	http://www.geocities.com/	0	19	21	0	15

^*^ Disclosure: the principal investigator (JS) is currently employed by Healthwise, but the review of this site (as well as all other sites that received high scores) was conducted by the external reviewer without influence from the author. Exclusion of this site from the analysis only changed the mean score by half of one percentage point.

## Appendix 2

### Kappa statistics for each criterion.

**Table A2:** Kappa statistics for each criterion

**Criterion**	**Agreement**	**Expected Agreement**	**Kappa**	***P***
**Process measures average**	**72.38%**	**56.57%**	**0.3422**	**Significant**
	Content generation explanation	66.67%	52.00%	0.3056	.0221
	Identification & disclosure	86.67%	50.00%	0.7333	< .001
	Referenced material	66.67%	51.56%	0.3119	.0303
	Peer review	66.67%	51.78%	0.3088	.0439
	Updating process	80.00%	76.67%	0.1429	.2071
	Content dating	66.67%	50.00%	0.3333	.0232
	Timely update	73.33%	64.00%	0.2593	.0742
	**Comprehensiveness average**	**79.45%**	**65.91%**	**0.4168**	**Significant**
	Screening	80.00%	55.56%	0.5500	.0013
	Glycemia tests	80.00%	58.89%	0.5135	< .001
	Exercise	90.00%	90.44%	-0.0465	.1711
	Acute episodes	73.33%	64.22%	0.2547	.0815
	Secondary diabetes	76.67%	49.11%	0.5415	< .001
	Foot care	73.33%	68.67%	0.1489	.0601
	Dyslipidemia	60.00%	52.89%	0.1509	.0588
	Smoking cessation	70.00%	49.11%	0.4105	.0087
	Nephropathy	96.67%	90.44%	0.6512	< .001
	Retinopathy	96.67%	90.44%	0.6512	< .001
	Immunization	93.33%	81.78%	0.6341	< .001
	Insulin administration	60.00%	42.22%	0.3077	.0098
	Oral medications	80.00%	60.67%	0.4915	.0032
	Glucose monitoring	80.00%	63.33%	0.4545	.0038
	Care of children	73.33%	64.22%	0.2547	.0815
	Gestational diabetes	86.67%	76.89%	0.4231	.0102
DCCT (Diabetes Control & Complications Trial) implications	76.67%	51.33%	0.5205	.0021
	UKPDS (United Kingdom Prevention of Diabetes Study) implications	86.67%	72.00%	0.5238	.0016
	Nutrition	96.67%	84.67%	0.7826	< .001
	Insulin/glucose explanation	76.67%	63.33%	0.3636	.0049
	Prevention	53.33%	44.67%	0.1566	.1412
	Psychological aspects	80.00%	57.78%	0.5263	.0018
	Neuropathy	90.00%	79.33%	0.5161	.0021
	Obesity	76.67%	70.00%	0.2222	.1103
**Accuracy average**	**81.33%**	**66.62%**	**0.3979**	**Significant**
	Type 1 vs Type 2	80.00%	76.00%	0.1667	.1361
	Secondary causes	96.67%	96.67%	0.0000	.5000
	Diagnostic tests	80.00%	50.00%	0.6000	< .001
	HbA1c test	76.67%	55.33%	0.4776	.0024
	Albumin tests	90.00%	79.33%	0.5161	.0021
	Cholesterol tests	76.67%	70.00%	0.2222	.1103
	Warning signs	60.00%	46.44%	0.2531	.0550
	Hypoglycemia prevention	73.33%	56.00%	0.3939	.0077
	Oral medications	90.00%	62.44%	0.7337	< .001
	Rezulin	90.00%	74.00%	0.6154	< .001
**Outcome composite average**	**80.00%**	**66.12%**	**0.4112**	**Significant**
**Overall average**	**78.70%**	**64.49%**	**0.3994**	**Significant**
